# Risk of Somatic Diagnoses in Specialist Health Care Among Norwegian-Born Youth and Young Adults with Immigrant Parents

**DOI:** 10.1007/s10903-025-01689-8

**Published:** 2025-05-16

**Authors:** Marte Karoline Raberg Kjollesdal, Naima Said Sheikh, Ylva Helland, Thor Indseth, Angela Susan Labberton

**Affiliations:** 1 Norwegian University of Life Sciences, Ås, Norway; 2 Norwegian Institute of Public Health, Oslo, Norway

**Keywords:** Descendant, Youth, Somatic health, Medical, Infectious, Diagnosis, Specialist health care

## Abstract

Knowledge about the somatic health of young adults born to immigrant parents is lacking. This study aims to assess the risk of receiving somatic diagnoses among Norwegian-born young adults with immigrant parents compared to their counterparts with two Norwegian-born parents. Data from Medical Birth Registry of Norway and Statistics Norway were linked to data from Norwegian Patient Registry on 37 diagnostic categories of somatic conditions given in specialist health care between 2008 and 2022. Norwegian-born individuals aged 16–30 years between 2008 and 2022 were included (N = 1 522 597). Hazard ratios (HR) of diagnoses by immigrant background were assessed by Cox regressions adjusted for sex, birth year, and parental education. Individuals with two immigrant parents had lower risk of receiving any somatic diagnosis [HR (95% confidence interval) 0.91 (0.90, 0.93)], as well as any infectious, medical or neurological diagnosis, than counterparts with two Norwegian-born parents. Those with an immigrant mother only had lower risk of any somatic diagnosis [HR 95% CI 0.94 (0.92, 0.95)] and of any medical or neurological diagnosis. These differences were not seen after adjustment for parental duration of residence and education. Those with an immigrant father only had higher risk of any somatic diagnosis [HR 95% CI 1.03 (1.02, 1.04)], as well as any infectious or neurological diagnosis. Norwegian-born young adults with two immigrant parents or an immigrant mother had lower risk than those with two Norwegian-born parents of receiving somatic diagnoses in specialist health care before adjustment for parental duration of residence and education, while those with an immigrant father only, had higher risk.

**Supplementary Information:**

The online version contains supplementary material available at 10.1007/s10903-025-01689-8.

## Background

As the number of immigrants has increased in Europe over the last decades, so too has the number of children born to immigrant parents. There is evidence that the health in this group is influenced by the immigrant status of their parents [1–3]. Poor living conditions, low parental education and health literacy, and a high burden of mental and somatic disease may negatively impact the health of children [4–6]. Moreover, poor access to health care caused by limited knowledge about the system and poor proficiency in the native language, may be barriers to timely care and good health among descendants of immigrants. In addition, differences in cultural perceptions of health and disease between parents and health personnel could influence the use of health care services and its quality. Importantly, inherent problems in the health care services provided to immigrants are reported, including lack of respect and inadequate use of interpreters [7, 8]. Although overall health does not seem to be worse among children with immigrant parents than others, some challenges are identified, including higher risk of obesity [1, 9–12], several types of infections, and skin diseases [1, 3].

Health consequences of childhood experiences may, or may not, persist into youth and young adulthood. When descendants of immigrants growing up in Europe enter youth and early adulthood, they will not have the same structural barriers, including language and lack of system knowledge, to health care, as their parents had. They might, however, have with them some cultural perceptions of health and disease from their family which are different that those of health care providers, as well as barriers associated with their phenotypic appearance, including experiences of discrimination within the health care system [13, 14]. While there is a body of evidence that European born youth and young adults with immigrant parents report more mental health issues than their native background counterparts [15, 16] but use health care for these conditions less frequently [17–19], there is a deficiency in the literature regarding the somatic health in this population. Three studies have reported on the risk of receiving a diagnosis for a somatic condition among youth with one immigrant parent [17, 20, 21]. The risk was not different between those with one immigrant parent only and those with two non-immigrant parents in Denmark [17], but slightly higher among those with an immigrant father (but not mother) compared to those with a native background in Finland [21]. In Norway, the risk of any somatic diagnoses, and a few selected diagnoses, in specialist health care was slightly lower among Norwegian-born youth aged 16–20 years with one or two immigrant parents, compared to those of native background [20].

In Norway, recent migration history started in the seventies and eighties, with working migrants coming mainly from Pakistan and Turkey, and considerable numbers of refugees coming from Vietnam, Iran, and Sri Lanka. In these groups, as well as in some newer groups of immigrants, larger numbers of descendants are now entering early adulthood. Knowledge about health in this group of youth and young adults is warranted to elucidate health needs and for planning of health services. Thus, we aim to leverage Norwegian register data to assess the risk of receiving somatic diagnoses in specialist health care among Norwegian-born youth and young adults (aged 16–30 years) with immigrant parents compared to their counterparts with two Norwegian-born parents. Further, we assess the role of socio-economic family factors (indicated by parental educational level at index person’s age of 10 years) in any differences found. Additionally, we discuss our findings in the light of previously published comparable estimates among younger children (aged 0–10 years) [1].

## Methods

### Study Population

**Fig. 1:**
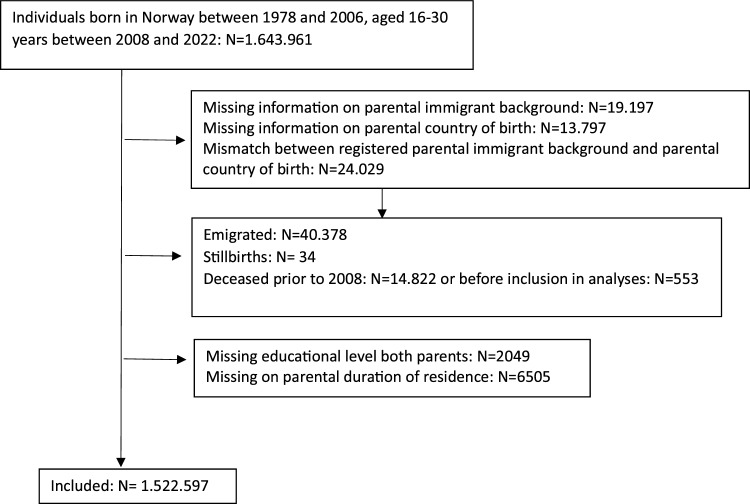
Flow chart

The study population included all individuals born in Norway between 1978 and 2006, and who were 16–30 years of age between 2008 and 2022 (N = 1.643.961). We excluded those who had missing information on parental immigrant background (N = 19.197), parental country of birth, (N = 13.797), or who were registered with a mismatch on parental immigrant background and parental country of birth (N = 24.029), those registered as emigrated (data on emigration year not available) (N = 40.378), as stillborn (N = 34), or as deceased prior to 2008 (N = 14.822) or before inclusion in analyses (N = 553), and those with missing on both parents’ educational level (N = 2049) or on parental duration of residence (N = 6505), leaving a sample of 1.522.597 individuals for the analysis (Fig. 1).

Data from the Medical Birth Registry of Norway were linked to data from the Norwegian Patient Registry (diagnoses from specialist health care), Statistics Norway (immigrant background, parental country of origin, duration of residence, and educational level), and Cause of Death Register, using the personal identification number.

### Measures

We included 37 diagnostic categories (following ICD-10 (International Classification of Disease)) from three domains of somatic health; infections, non-infectious medical conditions, and non-infectious neurological conditions registered in the Norwegian Patient Registry from 2008 (start of NPR) to 2022. The included ICD-10 codes are noted in Supplementary Table I. Individuals with a specific diagnosis registered in NPR at least once during the specified age and time frame were classified as having received the respective diagnosis. A dummy variable was constructed to indicate whether an individual had received any of the 37 included diagnoses. Among individuals with one or two immigrant parent(s), region of origin was classified according to national standards [22], based on parents’ country of birth (if different; mother’s); ‘EU/European Economic Area (EEA), Oceania, United States of America (USA) and Canada’ (hereafter called EU/EEA in text), ‘Europe outside the EU/EEA’, ‘Asia’, ‘Africa’ and ‘Latin America’.

The highest attained education of either parent registered in the year the index person was 10 years old was used as an indicator of SEP during up-bringing, and categorized into ‘primary school’ (started or completed/ ≤ 9 years), ‘upper secondary school’ (12 years), ‘university/university college, lower’ (completed a university/university college education of ≤ 4 years) and ‘university/university college, higher’ (completed a university/university college education of > 4 years). Duration of residence was measured as years in Norway at the time of the child’s birth for immigrant parents. Maternal duration of residence was used for those with two immigrant parents and those with one immigrant mother, and paternal duration of residence for those with an immigrant father only. Duration of residence were used as a continuous variable, with values > 25 set to 25. Norwegian-born parents were given the value 25.

### Analyses

Cox proportional hazard regressions were used to estimate the hazard ratio (HR) with 95% confidence intervals for each diagnosis among individuals with two immigrant parents, an immigrant mother and a Norwegian-born father, and an immigrant father and a Norwegian-born mother, as well as by region of origin compared individuals with two Norwegian-born parents. Based on a directed acyclic graph, we adjusted for sex and birth year as they could influence the risk of receiving a diagnosis. In addition, we included a model where we adjusted for parental duration of residence and education when index person was 10 years old, to estimate the association between parental immigrant background and risk of receiving a diagnosis which could not be accounted for by these factors (Supplementary Fig. 1). As a sensitivity analysis, we also performed a model only adjusting for parental education. Individuals were followed from 2008 until year of diagnosis, year of death, year of reaching the age of 30 years (if earlier than 2022) or 2022. Assumptions of proportional hazards were assessed by visual inspections of log–log plots and found reasonable. Analyses were performed using STATA 18 (StataCorp LLC, College Station, TX).

The study was approved by the Regional Ethics Committee South-East (REK 2019/1286), with exemption from informed consent from participants, due to the nature of national register data.

## Results

In the sample, 88.6% of the individuals had two Norwegian-born parents, 4.1% had two immigrant parents, 3.6% had an immigrant mother and a Norwegian-born father, and 3.8% had an immigrant father and Norwegian-born mother (Table 1). The majority of those with two immigrant parents had parents from Asia, whereas those with one immigrant parent most often had a parent from EU/EEA. Those with two immigrant parents were most likely to have been raised by parents with primary education only, while those with one immigrant parent had the highest percentage of parents with higher high education (Table 1). The mean duration of residence at child`s birth was 5.4 years for those with two immigrant parents, 8.6 years for those with an immigrant mother and 8.2 years for those with an immigrant father.

**Table 1:** Characteristics of the sample; Norwegian-born persons aged 16–30 years between 2008 and 2022

	N (%)*	Parental education (%)			
		Primary school	Upper secondary school	University/university college, lower	University/university college, higher
Two Norwegian born parents	1,354,054 (88.6)	9.5	47.5	31.7	11.3
Two immigrant parents	63,269 (4.1)	31.6	34.8	23.7	9.9
EU, EEA, Oceania, USA, Canada	5531 (8.7)	7.8	24.7	33.5	34.0
Europe except EU/EEA	6662 (10.5)	19.8	45.8	25.3	9.2
Asia	39,241 (62.0)	36.7	34.0	22.3	7.1
Africa	10,052 (15.9)	35.4	35.4	21.5	7.7
Latin America	1787 (2.8)	16.8	39.7	32.1	11.4
Immigrant mother only	54,244 (3.6)	7.7	33.4	36.2	22.8
EU, EEA, Oceania, USA, Canada	35,459 (65.4)	5.4	30.3	38.2	26.1
Europe except EU/EEA	2172 (4.0)	7.3	28.5	35.2	29.0
Asia	11,671 (21.5)	13.9	41.9	31.3	12.8
Africa	1743 (3.2)	8.8	37.0	36.7	17.6
Latin America	3203 (5.9)	9.7	37.4	31.7	21.2
Immigrant father only	57,535 (3.8)	10.6	34.1	37.0	18.4
EU, EEA, Oceania, USA, Canada	37,819 (65.7)	8.1	33.0	38.2	20.8
Europe except EU/EEA	1621 (2.8)	14.1	46.6	32.2	7.0
Asia	9073 (15.8)	18.6	37.6	30.9	13.0
Africa	5732 (10.0)	12.9	31.4	41.0	14.8
Latin America	3291 (5.7)	11.7	34.9	35.9	17.5

*Proportions in each region of origin given for those with two immigrant parents, an immigrant mother and an immigrant father separately

**Fig. 2:**
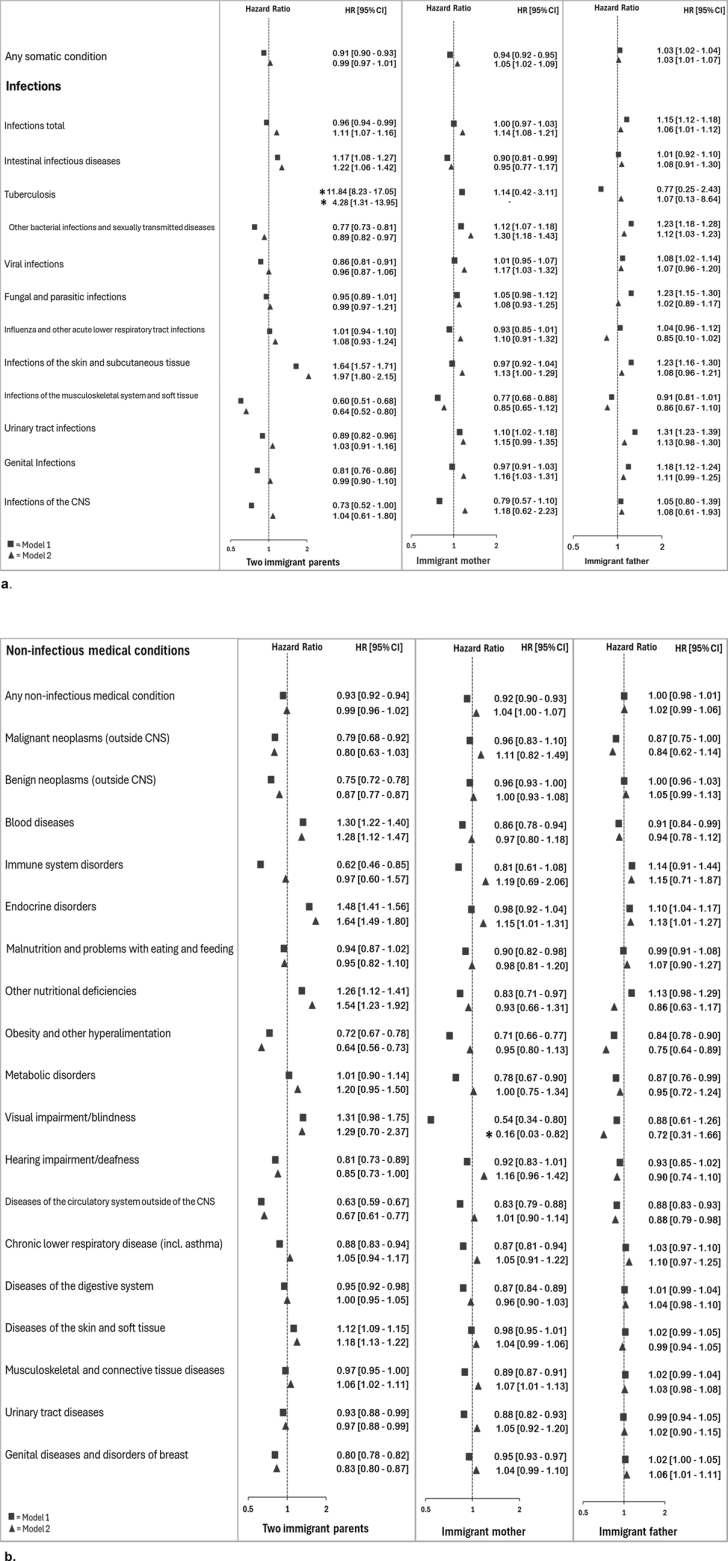

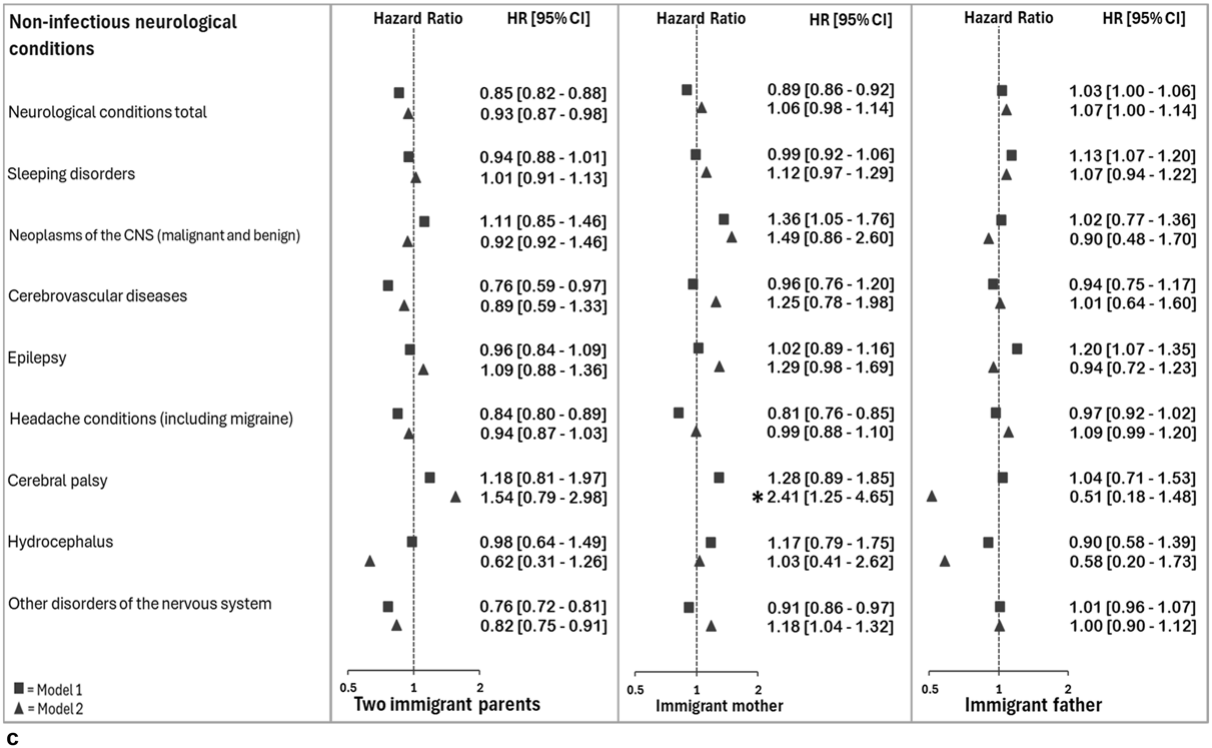
**a**–**c** Hazard Ratio (95% confidence interval) for diagnoses given in specialist health care between 2008 and 2022 among Norwegian-born persons 16–30 years having immigrant parents. Reference: two Norwegian-born parents. From Cox regressions, adjusted for year of birth and sex (model 1) and additionally for parental duration of residence at child’s birth and education when child’s age 10 (model 2). Estimates with asterisk (*) fall outside the range

Percentages who received different diagnoses by parental immigrant background are shown in Supplementary Table 2. The risk of a diagnosis of any somatic condition was lower among those with two immigrant parents [HR 95% CI 0.91 (0.90, 0.93)] or an immigrant mother only [HR 95% CI 0.94 (0.92, 0.95)], but slightly higher among those with an immigrant father [HR 95% CI 1.03 (1.02, 1.04)], than among those with two Norwegian-born parents (Fig. 2a).

### Infectious Diseases

The risk of any infectious diagnosis was lower among those with two immigrant parents, and higher among those with an immigrant father only, compared to those with two Norwegian-born parents (Fig. 2a). Individuals with two immigrant parents had lower risk than those with two Norwegian-born parents of receiving diagnoses of five types of infectious diseases, and higher risk of three. The HR was especially high for a tuberculosis diagnosis; however, the number of cases was low. There were few differences in risk of infectious diagnoses between those with an immigrant mother only and those with two Norwegian-born parents. Those with an immigrant father only had higher risk than those with two Norwegian-born parents for six out of eleven diagnostic groups for infections (Fig. 2a).

### Non-infectious Medical Conditions

The risk of a diagnosis of any non-infectious medical condition was lower among those with two immigrant parents and those with an immigrant mother only, than among those with two Norwegian-born parents, but slightly higher among those with and immigrant father (Fig. 2b). Those with two immigrant parents had lower risk of a diagnosis of ten conditions out of eighteen, and higher risk for a diagnosis of four conditions, than those with two Norwegian-born parents. The most pronounced differences were seen in low HR for diagnoses of immune system disorders, circulatory system diseases outside the CNS and obesity. Those with an immigrant mother had lower risk of thirteen diagnostic categories for non-infectious medical conditions, and not higher risk of any of the included categories. Those with an immigrant father had lower risk of a diagnosis of four conditions and a higher risk of a diagnosis of two conditions compared to those with two Norwegian-born parents.

### Non-infectious Neurological Conditions

Those with two immigrant parents or an immigrant mother only had a lower risk of a diagnosis of any non-infectious neurological condition than those with two Norwegian-born parents, whereas those with an immigrant father had higher risk (Fig. 2c). Among those with two immigrant parents, the risk was lower for three out of eight diagnostic categories compared to those with two Norwegian-born parents. Those with an immigrant mother had lower risk of a diagnoses of two condition, and higher for one, compared to those with two Norwegian-born parents. Among those with an immigrant father, the risk of two diagnostic categories was higher than among those with two Norwegian-born parents.

### Parental Region of Origin, Parental Duration of Residence and Education Level

The hazard ratios for the diagnoses by immigrant parents` region of origin are shown in Supplementary Table 3. Among those with two immigrant parents, the risk of any somatic disease was lower for those with parents from Asia and Latin America, compared to those with two Norwegian-born parents. The risk of any infectious diagnosis was lower among those with parents from Asia, but higher among those with parents from Africa and Latin America. For non-infectious medical and neurological conditions, the risk was lower than among youth and young adults with two Norwegian-born parents among those with parents from Asia or Africa. The pattern of lower or the same risk of a diagnosis of most conditions among those with an immigrant mother, and higher risk of some diagnoses among those with an immigrant father only, was relatively stable across regions of origin.

When analyses were adjusted for parental duration of residence and level of education during up-bringing, the risk of receiving several of the included diagnoses were no longer lower among those with two immigrant parents as compared to those with two Norwegian-born parents, and similar or even higher among those with an immigrant mother only (Fig. 2a, b, c). In sensitivity analyses adjusting for sex, birth year and parental education only, such changes in estimates were not seen.

## Discussion

Norwegian-born youth and young adults with two immigrant parents or with an immigrant mother only had lower risk than counterparts with two Norwegian-born parents in risk of receiving a diagnosis of any somatic condition, and of any non-infectious medical or neurological diagnosis, as well as of many single diagnostic categories. Those with an immigrant father only had higher risk of a diagnoses of any somatic condition, as well as of any infectious disease, any non-infectious neurological condition and several single conditions. Adjustment for parental duration of residence significantly attenuated the results for those with two immigrant parents or an immigrant mother only.

In line with our findings, a Finnish study demonstrated that the risk of receiving a diagnosis of any somatic condition was slightly higher among those with an immigrant father, but not immigrant mother, compared to those with a native background [21]. In Denmark, the risk was not different between those with one immigrant parent and those with Danish background [17]. Our findings are also in line with a recent Norwegian study [20], showing lower risk of receiving any somatic diagnosis in specialist health care in general among Norwegian-born youth aged 16–20 years old having two or one immigrant parents. A lower risk of a diagnosis asthma among those with immigrant parents, was also reflected in our findings of lower risk of chronic lower respiratory disease (including asthma) among those with two immigrant parents or an immigrant mother only. However, their findings of a lower risk of a diabetes type 1 diagnosis among youth with two or one immigrant parent, and of an epilepsy diagnosis among those with two immigrant parents, is contrary to our results. Our inclusion of a wider age-group and broad diagnosis categories may explain the differences.

The lower risk of receiving many diagnoses among those with two immigrant parents or an immigrant mother may have several explanations. Firstly, it could be that these youth and young adults have better health than those with two Norwegian-born parents. Immigrants are often healthier than the non-immigrant population, at least in the first years of residence, commonly referred to as the `healthy migrant effect` [23]. There is evidence suggesting that the healthy migrant effect is transferred to descendants through cultural environment and lifestyle, or through genetic transmission, albeit it`s being diluted over generations [20]. If the effect is also seen among descendants, the premise of healthy selection is challenged [23], unless it is closely related to genetics. The lower risk of receiving a diagnosis across various domains of somatic health, as well as differences between those having an immigrant father and an immigrant mother, does not point toward the importance of certain genetic make-ups. Notably, the healthy migrant effect generally decreases over time, in part due to disadvantaged living conditions and stressful life situations related to being an immigrant [24], and this change in health among immigrants may also negatively affect their children. Moreover, descendants of immigrants are described as experiencing a health convergence towards the health of those with native-born parents [17, 25], through adoption of health-related behavior of the general population, and stressors related to immigrant background, including discrimination. Secondly, youth and young adults with immigrant parents may have the same health as counterparts with Norwegian-born parents, but lower chance of being diagnosed in specialist health care. In Norway, youth can seek health care without the involvement of guardians from the age of 16, and those born in Norway have no special barriers to seeking health care related to lack of language or knowledge of the system. Still, parents may impact their children’s use of services, both directly through practical help or emotional support, and indirectly through perceptions of health, disease and health care seeking behavior, as well as low health literacy during the upbringing. It might also be that descendants face discrimination in the health care system based on being (incorrectly) identified as an immigrant and thus receive health care of lower quality than others. Discrimination of immigrants within health care is reported both in the form of stigmatization and lack of respect and cultural understanding from health care personnel, as well as inadequate use of interpreters and lack access to information [7, 8, 13, 14, 26, 27]. Thirdly, descendants may have the same health as those with two Norwegian-born parents, but there is overdiagnosis among those with Norwegian-born parents. Mothers are often the primary care giver in families and the one responsible for seeking health care for the children. Thus, the fact that those with a Norwegian-born mother and an immigrant father had higher risk of a diagnosis of many somatic conditions, points towards the importance of barriers to health care among those with an immigrant mother. Also, the attenuation of estimates when adjusting for maternal duration of residence supports the hypothesis that barriers related to maternal system knowledge and language proficiency in childhood play a role. It also downplays the possibility that children with one or two immigrant parents should be of better health than others.

### Comparisons to Younger Children

The risks of somatic diagnoses in specialist health care among younger Norwegian-born children (0–10 years) with immigrant parents have previously been reported [1]. Younger children with immigrant parents had higher risk than those with two Norwegian-born parents of a diagnosis of any somatic condition, and of diagnoses of infectious diseases. This was not seen among the youth and young adults. It thus seems that use of specialist health services for somatic condition in general, and of infections especially, among Norwegian-born with immigrant parents is converging towards that of counterparts with two Norwegian-born parents with older age. For the various non-infectious conditions, the picture was largely the same in both age-groups. One striking difference was a higher risk of an obesity diagnosis among younger children with immigrant parents compared to children with two Norwegian-born parents, but a lower risk of an obesity diagnosis among youth and young adults with immigrant parents. This may reflect changes in differences in overweight and obesity by immigrant background with age. An older Norwegian article has demonstrated low BMI levels among youth from Asia and Africa [28], although children from the same regions have been shown to have high levels of overweight [9]. It might also be that youth and young adults with an immigrant background are less likely to be diagnosed and/or treated for obesity in the specialist health services than their counterparts with two Norwegian-born parents, whereas younger children are as likely as, or more than, children with two Norwegian-born parents to be diagnosed.

### Strengths and Limitations

Strengths of the study include use of register data with national coverage over a range of diagnoses, and with linkage to immigrant background and parental educational level. Available diagnoses include only those given in specialist care. They may thus reflect more serious or further developed conditions than those treated in primary care. Results may to some extent also reflect use of primary care, as some of the diagnoses given in specialist health care could have been prevented if treated timely in primary care. Moreover, in the Norwegian health care system the general practitioner is the gatekeeper to specialist health services. We did not have access to data on reason for migration, which would have been useful, as it may be related to both health and barriers to and within health care.

### New Contributions to the Literature

Few available studies report on somatic health among descendants of immigrants in early adulthood in Europe, and our article provides an important contribution to start to better understand this topic.

## Conclusions

In general, Norwegian-born youth and young adults with two immigrant parents or one immigrant mother only had lower risk than those with two Norwegian-born parents of receiving somatic diagnoses in specialist health care, while those with an immigrant father only had higher risk. The importance of various barriers to health care use among young adults with immigrant parents should be further examined.

## Supplementary Information

Below is the link to the electronic supplementary material.

Supplementary file1 (DOCX 170 KB)

Supplementary file2 (DOCX 127 KB)

## Data Availability

The datasets generated and analyzed during this study are not publicly available due to data regulations but can be obtained from registry owners.
