# Supplementary material for: Risk of Somatic Diagnoses in Specialist Health Care Among Norwegian-Born Youth and Young Adults with Immigrant Parents
Source: J Immigr Minor Health. 2025 May 16;27(4):586–94. doi: 10.1007/s10903-025-01689-8 (PMC12255552; doi:10.1007/s10903-025-01689-8)
Supplement: Supplementary file 1 — Supplementary file1 (DOCX 170 KB) [file 10903_2025_1689_MOESM1_ESM.docx]

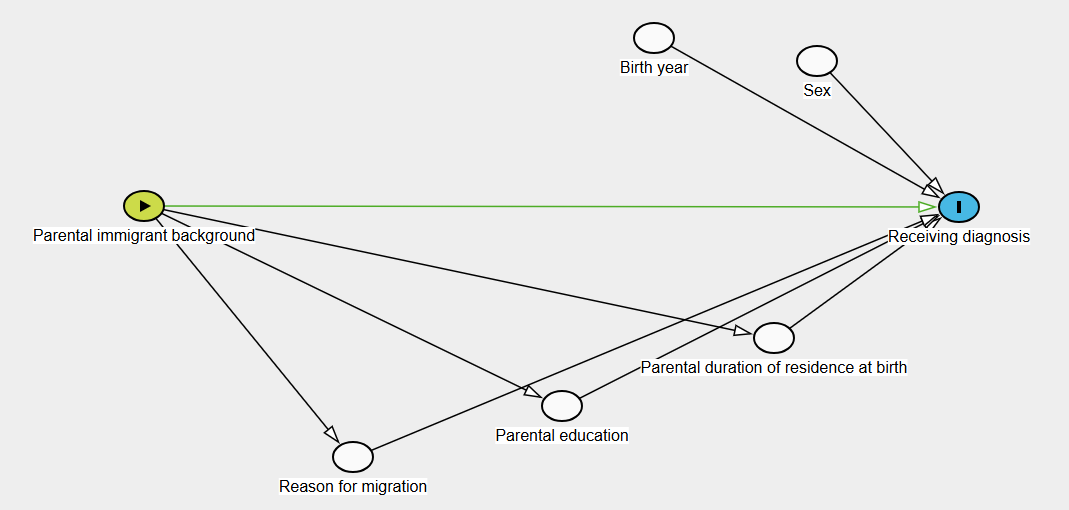


*

**Supplementary Figure 1**. Direct acyclic graph for the relationship between parental immigrant background and receiving a diagnosis in specialist health care among Norwegian born youth and young adults.

*Data on reason for migration was not available for analyses.
