# Supplementary material for: Risk of Somatic Diagnoses in Specialist Health Care Among Norwegian-Born Youth and Young Adults with Immigrant Parents
Source: J Immigr Minor Health. 2025 May 16;27(4):586–94. doi: 10.1007/s10903-025-01689-8 (PMC12255552; doi:10.1007/s10903-025-01689-8)
Supplement: Supplementary file 2 — Supplementary file2 (DOCX 127 KB) [file 10903_2025_1689_MOESM2_ESM.docx]

**Supplementary tables**

| **Supplementary table 1.** Categorization of diseases | |
| --- | --- |
| **CATEGORY** | **ICD-10 CODES** |
| **Infections** |  |
| Intestinal infectious diseases | A00–A09 |
| Tuberculosis | A15–A19 |
| Other bacterial infections and sexually transmitted diseases | A20–A79, B95, B96, B98 |
| Viral infections | A80–B34, B97 |
| Fungal and parasitic infections | B35–B89 |
| Influenza and other acute lower respiratory tract infections | J09–J22 |
| Infections of the skin and subcutaneous tissue | L00–L08 |
| Infections of the musculoskeletal system and soft tissue^a^ | M00–M03, M60, M65, M86 |
| Urinary tract infections^a^ | N10, N12, N30, N34 |
| Genital infections^a^ | N41, N45, N49, N61, N70 – N77 |
| Infections of the CNS^b^ | G00–G09 |
| **Non-infectious medical conditions** |  |
| Malignant neoplasms outside of the CNS^b^ | C00–C69, C73–C97 |
| Benign neoplasms and carcinoma in situ, outside of the CNS^b^ | D00–D31, D34–D41, D44–D48 |
| Blood diseases | D50–D77 |
| Immune system disorders | D80–D89 |
| Endocrine disorders | E00–E35 |
| Malnutrition and problems with eating and feeding | E40–E46, R13, R63–R64 |
| Other nutritional deficiencies | E50–E64 |
| Obesity and other hyperalimentation | E65–E68 |
| Metabolic disorders | E70–E72, E74–E85, E88, E89 |
| Visual impairment/blindness | H54 |
| Hearing impairment/deafness | H90–H91 |
| Diseases of the circulatory system outside of the CNS^b, c^ | I00–I52, I70–I79, I81, I82 |
| Chronic lower respiratory disease (including asthma) | J40–J47 |
| Diseases of the digestive system^d^ | K20–K31, K40–K64, K70–K93 |
| Diseases of the skin and soft tissue | L10–L99 |
| Diseases of the musculoskeletal system and connective tissue | M05–M54, M61–M63, M66–M85, M87–M99 |
| Urinary tract diseases | N00–N08, N11, N13–N29, N31–N33, N35–N39 |
| Genital diseases and disorders of breast | N40, N42–N44, N46–N48, N50–N60, N62–N64, N80–N99 |
| **Non-infectious neurological conditions** |  |
| Sleeping disorders | G47, F51 |
| Neoplasms of the CNS^b^ (malignant and benign) | C70–C72, D32–D33, D42–D43 |
| Cerebrovascular diseases | I60–I69, G45, G46 |
| Epilepsy | G40–G41 |
| Headache conditions (including migraine) | G43–G44, R51 |
| Cerebral palsy | G80 |
| Hydrocephalus | G91 |
| Other disorders of the nervous system | G10–G37, G50–G73, G81–G90, G92–G99 |

| **Supplementary table 2.** Number (per 1000) with diagnosis given in specialist health care between 2008 and 2022 among Norwegian-born persons 16-30 year by parental immigrant background | | | | |
| --- | --- | --- | --- | --- |
|  | Two Norwegian-born parents  N=1 354 054 | Two immigrant parents  N=61 391 | Immigrant mother only  N=52 958 | Immigrant father only  N=54 194 |
| **Any somatic condition** | 736 680 (544.1) | 32 318 (526.4) | 27 028 (510.4) | 30 276 (558.7) |
| **Infections** |  |  |  |  |
| Infections total | 195 215 (144.2) | 8809 (143.5) | 7321 (138.2) | 8848 (163.3) |
| Intestinal infectious diseases | 16 596 (12.2) | 1064 (17.3) | 627 (11.8) | 707 (13.0) |
| Tuberculosis | 124 (0.1) | 65 (1.1) | 8 (0.2) | 6 (0.1) |
| Other bacterial infections and sexually transmitted diseases | 51 913 (38.3) | 1669 (27.2) | 2050 (38.7) | 2429 (44.8) |
| Viral infections | 37 336 (27.6) | 1573 (25.6) | 1470 (27.8) | 1673 (30.9) |
| Fungal and parasitic infections | 22 768 (16.8) | 1045 (17.0) | 891 (16.8) | 1150 (21.2) |
| Influenza and other acute lower respiratory tract infections | 18634 (13.8) | 930 (15.1) | 693 (13.1) | 809 (14.9) |
| Infections of the skin and subcutaneous tissue | 33 562 (24.8) | 2502 (40.8) | 1252 (23.6) | 1679 (31.0) |
| Infections of the musculoskeletal system and soft tissue | 10 107 (7.5) | 269 (4.4) | 296 (5.6) | 366 (6.8) |
| Urinary tract infections | 22 059 (16.3) | 802 (13.1) | 847 (16.0) | 1089 (20.1) |
| Genital Infections | 34 966 (25.8) | 1108 (18.0) | 1174 (22.2) | 1567 (28.9) |
| Infections of the CNS | 1627 (1.2) | 49 (0.8) | 52 (1.0) | 75 (1.4) |
| **Non-infectious medical conditions** |  |  |  |  |
| Any non-infectious medical condition | 659 056 (487.2) | 28 823 (469.5) | 23 875 (450.8) | 26 692 (492.5) |
| Malignant neoplasms (outside CNS) | 8352 (6.2) | 260 (4.2) | 293 (5.5) | 276 (5.7) |
| Benign neoplasms (outside CNS) | 112 058 (82.8) | 3487 (56.8) | 3822 (72.2) | 4276 (80.7) |
| Blood diseases | 19 047 (14.1) | 1183 (19.3) | 652 (12.3) | 714 (13.2) |
| Immune system disorders | 2312 (1.7) | 72 (1.2) | 71 (1.3) | 97 (1.7) |
| Endocrine disorders | 45 703 (33.8) | 2445 (39.8) | 1572 (29.7) | 1888 (34.0) |
| Malnutrition and problems with eating and feeding | 16 521 (12.2) | 951 (15.5) | 637 (12.0) | 748 (13.8) |
| Other nutritional deficiencies | 5783 (4.3) | 470 (7.7) | 236 (4.5) | 296 (5.5) |
| Obesity and other hyperalimentation | 28 780 (21.3) | 1152 (18.8) | 776 (14.7) | 1075 (19.8) |
| Metabolic disorders | 9413 (7.0) | 403 (6.6) | 281 (5.3) | 332 (6.1) |
| Visual impairment/blindness | 1203 (0.9) | 71 (1.2) | 28 (0.5) | 39 (0.7) |
| Hearing impairment/deafness | 19 335 (14.3) | 792 (12.9) | 706 (13.3) | 731 (13.5) |
| Diseases of the circulatory system outside of the CNS | 47 400 (35.0) | 1288 (21.0) | 1462 (27.6) | 1644 (30.3) |
| Chronic lower respiratory disease (including asthma) | 48 547 (35.9) | 2409 (39.2) | 1838 (34.7) | 2207 (40.7) |
| Diseases of the digestive system | 150 204 (110.9) | 6316 (102.9) | 4889 (92.3) | 6099 (112.5) |
| Diseases of the skin and soft tissue | 191 648 (141.5) | 10 675 (173.9) | 7590 (143.3) | 8220 (151.7) |
| Diseases of the musculoskeletal system and connective tissue | 233 198 (172.2) | 9708 (158.1) | 7742 (146.2) | 9323 (172.0) |
| Urinary tract diseases | 37 660 (27.8) | 1539 (25.1) | 1228 (23.2) | 1451 (26.8) |
| Gential diseases and disorders of breast | 192 461 (142.1) | 6511 (106.0) | 6562 (123.9) | 7657 (141.3) |
| **Non-infectious neurological conditions** |  |  |  |  |
| Neurological conditions total | 126 577 (93.5) | 4772 (77.7) | 4272 (80.7) | 5263 (97.1) |
| Sleeping disorders | 27 378 (20.2) | 1136 (18.5) | 984 (18.6) | 1269 (23.4) |
| Neoplasms of the CNS (malignant and benign) | 1923 (1.4) | 82 (1.3) | 92 (1.7) | 67 (1.2) |
| Cerebrovascular diseases | 2665 (2.0) | 91 (1.5) | 95 (1.8) | 101 (1.9) |
| Epilepsy | 14 470 (10.7) | 649 (10.6) | 530 (10.0) | 665 (12.3) |
| Headache conditions (including migraine) | 54 605 (40.3) | 2003 (32.6) | 1738 (32.8) | 2294 (40.5) |
| Cerebral palsy | 2985 (2.2) | 161 (2.6) | 128 (2.4) | 127 (2.3) |
| Hydrocephalus | 1308 (1.0) | 60 (1.0) | 50 (0.9) | 49 (0.9) |
| Other disorders of the nervous system | 44 476 (32.8) | 1509 (24.6) | 1480 (27.9) | 1788 (33.0) |

| **Supplementary table 3.** Hazard Ratio (95% confidence interval) for diagnoses given in specialist health care between 2008 and 2022 among Norwegian-born persons 16-30 years having immigrant parents, by parental region of origin. Reference: Two Norwegian-born parents. From Cox regressions, adjusted for year of birth and sex (model 1) and additionally for parental duration of residence at child`s birth and education when child was 10 years old (model 2). | | | | | | | | | | |
| --- | --- | --- | --- | --- | --- | --- | --- | --- | --- | --- |
|  | EU, EEA, USA, Canada, Oceania | | Europe except EU/EEA | | Asia | | Africa | | Latin America | |
|  | Model 1 | Model 2 | Model 1 | Model 2 | Model 1 | Model 2 | Model 1 | Model 2 | Model 1 | Model 2 |
| TWO IMMIGRANT PARENTS |  |  |  |  |  |  |  |  |  |  |
| **Any somatic condition** | 1.00  (0.91, 0.96) | 1.09  (1.01, 1.17) | 0.98  (0.89, 1.07) | 1.11  (1.03, 1.20) | 0.86  (0.83, 0.89) | 0.97  (0.91, 1.03) | 0.96  (0.88, 1.04) | 0.95  (0.88, 1.01) | 0.91  (0.85, 0.97) | 1.19  (1.09, 1.30) |
| **Infections** |  |  |  |  |  |  |  |  |  |  |
| Infections total | 1.03  (0.95, 1.12) | 1.14  (1.00, 1.29) | 0.97  (0.89, 1.05) | 1.06  (0.93, 1.21) | 0.91  (0.88, 0.94) | 0.98  (0.89, 1.09) | 1.09  (1.01, 1.15) | 1.17  (1.04, 1.31) | 1.22  (1.09, 1.37) | 1.3  (1.15, 1.55) |
| Intestinal infectious diseases | 1.17  (0.89, 1.54) | 1.29  (0.86, 1.96) | 1.27  (0.99, 1.65) | 1.30  (0.85, 2.01) | 1.14  (1.04, 1.26) | 1.13  (0.82, 1.57) | 1.18  (0.96, 1.45) | 1.18  (0.80, 1.73) | 1.46  (1.01, 2.10) | 1.52  (1.15, 2.46) |
| Tuberculosis | - | - | - | - | 12.69  (8.40, 19.17) | 4.36  (0.82, 23.15) | 27.85 (15.46, 50.17) | 9.14  (1.58, 52.94) | - | - |
| Other bacterial infections and sexually transmitted diseases | 0.94  (0.80, 1.10) | 0.84  (0.77, 1.06) | 0.85  (0.72, 1.01) | 0.79  (0.59, 1.06) | 0.66  (0.61, 0.70) | 0.68  (0.50, 0.78) | 1.01  (0.90, 1.14) | 0.96  (0.75, 1.23) | 1.32  (1.08, 1.61) | 1.22  (0.91, 1.64) |
| Viral infections | 1.00  (0.83, 1.21) | 1.08  (0.81, 1.45) | 0.65  (0.52, 0.81) | 0.70  (0.50, 0.97) | 0.88  (0.82, 0.95) | 0.94  (0.75, 1.19) | 0.74  (0.63, 0.87) | 0.79  (0.60, 1.05) | 0.96  (0.71, 1.29) | 1.03  (0.71, 1.49) |
| Fungal and parasitic infections | 1.11 (0.90,1.38) | 1.04  (0.75, 1.45) | 1.05  (0.86, 1.28) | 1.02  (0.72, 1.44) | 0.85  (0.78, 0.93) | 0.84  (0.64, 1.11) | 1.10  (0.94, 1.29) | 1.08  (0.80, 1.48) | 1.37  (1.24, 1.83) | 1.32  (0.89, 1.95) |
| Influenza and other acute lower respiratory tract infections | 0.58  (0.40, 0.83) | 0.82  (0.51, 1.30) | 0.87  (0.65, 1.16) | 1.14  (0.74, 1.77) | 1.02  (0.92, 1.12) | 1.25  (0.92, 1.71) | 1.27  (1.06, 1.54) | 1.59  (1.11, 1.28) | 1.26  (0.87, 1.83) | 1.66  (1.03, 1.95) |
| Infections of the skin and subcutaneous tissue | 1.14  (0.95 ,1.37) | 1.33  (1.04, 1.71) | 1.37  (1.17, 1.61) | 1.54  (1.20, 1.97) | 1.69  (1.60, 1.79) | 1.84  (1.54, 2.19) | 1.91  (1.71, 2.13) | 2.09  (1.70, 2.57) | 1.33  (1.03, 1.72) | 1.50  (1.10, 2.04) |
| Infections of the musculoskeletal system and soft tissue | 0.73  (0.47, 1.11) | 0.98  (0.51, 1.90) | 0.65  (0.42, 1.01) | 0.81  (0.39, 1.67) | 0.57  (0.48, 0.67) | 0.66  (0.39, 1.14) | 0.56  (0.39, 0.81) | 0.66  (0.35, 1.27) | 0.82  (0.46, 1.49) | 1.03  (0.47, 2.28) |
| Urinary tract infections | 1.23  (0.98, 1.53) | 1.19  (0.82, 1.73) | 1.02  (0.80, 1.29) | 1.01  (0.67, 1.54) | 0.72  (0.65, 0.80) | 0.73  (0.53, 1.02) | 1.16  (0.98, 1.39) | 1.17  (0.82, 1.67) | 1.63  (1.23, 2.17) | 1.62  (1.06, 2.47) |
| Genital Infections | 1.13  (0.94, 1.35) | 1.61  (1.21, 2.15) | 0.97  (0.80, 1.17) | 1.38  (1.00, 1.90) | 0.71  (0.65, 0.77) | 0.95  (0.75, 1.22) | 0.89  (0.76, 1.04) | 1.22  (0.92, 1.63) | 1.29  (1.00, 1.65) | 1.79  (1.27, 2.53) |
| Infections of the CNS | - | - | 1.14  (0.49, 2.87) | 1.15  (0.20, 6.54) | 0.60  (0.38, 0.93) | 0.60  (0.14, 2.38) | 1.29  (0.67, 2.48) | 1.23  (0.26, 5.79) | - | - |
| **Non-infectious medical conditions** |  |  |  |  |  |  |  |  |  |  |
| Any non-infectious medical condition | 0.93  (0.88, 0.98) | 1.04  (0.96, 1.13) | 1.04  (0.99, 1.09) | 1.11  (1.02, 1.20) | 0.93  (0.91, 0.94) | 0.97  (0.91, 1.03) | 0.85  (0.82, 0.89) | 0.89  (0.83, 0.96) | 1.05  (0.98, 1.14) | 1.14  (1.03, 1.25) |
| Malignant neoplasms (outside CNS) | 1.11  (0.72, 1.70) | 1.08  (0.67, 2.84) | 1.20  (0.70, 1.73) | 1.43  (0.64, 3.18) | 0.66  (0.54, 0.80) | 0.83  (0.44, 1.56) | 0.90  (0.61, 1.31) | 1.14  (0.55, 2.35) | 1.44  (0.84, 2.48) | 1.82  (1.03, 1.25) |
| Benign neoplasms (outside CNS) | 1.00  (0.89, 1.11) | 1.30  (1.08, 1.55) | 0.99  (0.89, 1.11) | 1.38  (1.14, 1.67) | 0.71  (0.68, 0.74) | 0.96  (0.83, 1.12) | 0.61  (0.55, 0.68) | 0.83  (0.69, 1.00) | 0.84  (0.70, 1.00) | 1.33  (0.90, 1.42) |
| Blood diseases | 1.10  (0.76, 1.31) | 1.12  (0.77, 1.64) | 1.04  (0.80, 1.34) | 1.03  (0.69, 1.52) | 1.32  (1.21, 1.44) | 1.25  (0.94, 1.66) | 1.70  (1.45, 1.99) | 1.62  (1.18, 2.24) | 0.88  (0.57, 1.34) | 0.89  (0.54, 1.48) |
| Immune system disorders | - | - | - | - | 0.74  (0.52, 1.05) | 1.13  (0.35, 3.67) | 0.44  (0.16, 1.16) | 0.68  (0.15, 3.12) | - | - |
| Endocrine disorders | 1.34  (0.94, 1.36) | 1.57  (1.21, 2.03) | 1.48  (1.26, 1.75) | 1.92  (1.48, 2.50) | 1.61  (1.52, 1.70) | 1.96  (1.61, 2.37) | 1.09  (0.94, 1.26) | 1.35  (1.06, 1.72) | 1.56  (1.23, 1.98) | 2.02  (1.49, 2.74) |
| Malnutrition and problems with eating and feeding | 0.79  (0.58, 1.09) | 0.79  (0.51, 1.23) | 0.83  (0.63, 1.09) | 0.73  (0.47, 1.14) | 0.93  (0.84, 1.03) | 0.80  (0.58, 1.11) | 1.15  (0.95, 1.39) | 1.00  (0.69, 1.45) | 1.03  (0.68, 1.57) | 0.95  (0.56, 1.60) |
| Other nutritional deficiencies | 0.77  (0.46, 1.30) | 1.48  (0.79, 2.80) | 0.51  (0.30, 0.89) | 0.87  (0.44, 1.72) | 1.46  (1.27 1.67) | 2.16  (1.48, 3.16) | 1.40  (1.06, 1.83) | 2.15  (1.35, 3.43) | - | - |
| Obesity and other hyperalimentation | 0.50  (0.36, 0.69) | 0.90  (0.59, 1.39) | 0.98  (0.78, 1.23) | 1.24  (0.83, 1.87) | 0.66  (0.60, 0.73) | 0.70  (0.51, 0.95) | 0.77  (0.63, 0.94) | 0.83  (0.58, 1.21) | 1.60  (1.23, 2.08) | 2.15  (1.44, 3.72) |
| Metabolic disorders | 0.36  (0.18, 0.72) | 0.63  (0.28, 1.40) | 0.92  (0.60, 1.42) | 1.58  (0.83, 3.02) | 1.16 (1.01,1.33) | 1.82  (1.16, 2.86) | 0.69  (0.47, 1.01) | 1.11  (0.61, 2.00) | 1.26  (0.73, 2.17) | 2.11  (1.05, 4.26) |
| Visual impairment/blindness | - | - | 1.23  (0.46, 3.28) | 1.25  (0.26, 6.01) | 1.35  (0.95, 1.92) | 1.27  (0.40, 4.04 | 1.73  (0.90, 3.34) | 1.65  (0.44, 6.18) | - | - |
| Hearing impairment/deafness | 0.99  (0.73, 1.33) | 1.13  (0.70, 1.80) | 0.77  (0.55, 1.06) | 0.82  (0.48, 1.40) | 0.82  (0.73, 0.93) | 0.85  (0.58, 1.25) | 0.65  (0.49, 0.86) | 0.68  (0.42, 1.09) | 0.76  (0.46, 1.26) | 0.82  (0.44, 1.54) |
| Diseases of the circulatory system outside of the CNS | 0.34  (0.61, 0.90) | 0.84  (0.62, 1.14) | 0.68  (0.56, 0.83) | 0.73  (0.53, 1.02) | 0.58 (0.54,0.63) | 0.61  (0.47, 0.78) | 0.69  (0.59, 0.80) | 0.73  (0.54, 0.97) | 0.92  (0.71, 1.19) | 1.00  (0.70, 1.43) |
| Chronic lower respiratory disease (including asthma) | 0.78  (0.62, 0.99) | 0.76  (0.54, 1.07) | 0.74  (0.59, 0.94) | 0.71  (0.49, 1.01) | 0.91  (0.84, 0.98) | 0.86  (0.66, 1.13) | 0.79  (0.66, 0.95) | 0.75  (0.55, 1.03) | 1.32  (1.00, 1.75) | 1.27  (0.87, 1.85) |
| Diseases of the digestive system | 0.90  (0.82, 1.00) | 0.99  (0.85, 1.15) | 1.12  (1.03, 1.23) | 1.09  (0.93, 1.27) | 0.90  (0.87, 0.94) | 0.84  (0.75, 0.95) | 1.03  (0.96, 1.10) | 0.97  (0.84, 1.12) | 1.19  (1.04, 1.36) | 1.19  (0.99, 1.42) |
| Diseases of the skin and soft tissue | 0.99  (0.91, 1.08) | 1.14  (1.01, 1.28) | 1.16  (1.08, 1.24) | 1.31  (1.17, 1.48) | 1.18  (1.15, 1.21) | 1.32  (1.20, 1.44) | 0.89  (0.83, 0.95) | 1.00  (0.89, 1.12) | 1.16  (1.03, 1.31) | 1.31  (1.13, 1.52) |
| Diseases of the musculoskeletal system and connective tissue | 0.93  (0.86, 1.01) | 1.11  (0.98, 1.25) | 1.14  (1.06, 1.23) | 1.24  (1.09, 1.41) | 0.94  (0.92, 0.97) | 0.98  (0.89, 1.08) | 1.01  (0.95, 1.07) | 1.05  (0.94, 1.18) | 1.09  (0.97, 1.23) | 1.21  (1.04, 1.40) |
| Urinary tract diseases | 0.86  (0.70, 1.06) | 0.95  (0.88, 1.02) | 1.08  (0.90, 1.30) | 0.93  (0.66, 1.31) | 0.92  (0.86, 0.99) | 1.11  (0.97, 1.27) | 0.88  (0.75, 1.03) | 0.99  (0.83, 1.17) | 1.18  (0.91, 1.54) | 1.20  (0.97, 1.49) |
| Genital diseases and disorders of breast | 0.95  (0.88, 1.04) | 1.08  (0.95, 1.24) | 0.92  (0.85, 1.00) | 0.99  (0.86, 1.14) | 0.77  (0.75, 0.80) | 0.80  (0.72, 0.89) | 0.75  (0.70, 0.81) | 0.79  (0.69, 0.90) | 1.02  (0.90, 1.15) | 1.10  (0.94, 1.30) |
| **Non-infectious neurological conditions** |  |  |  |  |  |  |  |  |  |  |
| Neurological conditions total | 0.98  (0.88, 1.10) | 1.32  (1.11, 1.56) | 0.95  (0.86, 1.06) | 1.16  (0.96, 1.38) | 0.82  (0.79, 0.86) | 0.93  (0.81, 1.07) | 0.83  (0.76, 0.91) | 0.96  (0.82, 1.13) | 0.93  (0.79, 1.10) | 1.14  (0.92, 1.41) |
| Sleeping disorders | 0.98  (0.79, 1.22) | 1.56  (1.14, 2.14) | 0.76  (0.59, 0.96) | 1.09  (0.76, 1.55) | 0.96  (0.88, 1.03) | 1.25  (0.97, 1.60) | 0.89  (0.75, 1.06) | 1.20  (0.89, 1.61) | 1.32  (1.00, 1.74) | 1.91  (1.33, 2.75) |
| Neoplasms of the CNS (malignant and benign) | 1.51  (0.68, 3.36) | 1.68  (0.45, 6.31) | 1.52  (0.68, 3.39) | 1.75  (0.41, 7.41) | 1.00  (0.70, 1.42) | 1.15  (0.37, 3.56) | 1.05  (0.50, 2.32) | 1.21  (0.32, 4.63) | - | - |
| Cerebrovascular diseases | 0.82  (0.37, 1.83) | 2.72  (0.94, 7.92) | 0.56  (0.21, 1.50) | 2.07  (0.56, 7.65) | 0.67  (0.49, 0.91) | 2.05  (0.91, 4.61) | 1.08  (0.63, 1.86) | 3.50  (1.35, 9.11) | - | - |
| Epilepsy | 0.76  (0.46, 1.24) | 0.85  (0.42, 1.70) | 0.89  (0.57, 1.39) | 0.90  (0.44, 1.84) | 0.90  (0.77, 1.06) | 0.89  (0.52, 1.50) | 1.34  (1.02, 1.78) | 1.32  (0.73, 2.40) | 1.01  0.53, 1.94) | 1.05  (0.46, 1.24) |
| Headache conditions (including migraine) | 0.86  (0.72, 1.02) | 1.15  (0.89, 1.47) | 1.05  (0.90, 1.21) | 1.26  (0.98, 1.62) | 0.83  (0.77, 0.88) | 0.93  (0.76, 1.13) | 0.79  (0.69, 0.90) | 0.90  (0.72, 1.14) | 0.90  (0.70, 1.16) | 1.10  (0.80, 1.50) |
| Cerebral palsy | 0.93  (0.23, 3.73) | 2.49  (0.40, 5.35) | 1.63  (0.15, 18.17) | 0.51  (0.07, 3.66) | 1.25  (0.80, 1.95) | 3.22  (0.85, 12.17) | 1.60  (0.66, 3.85) | 4.25  (0.88, 20.41) | - | - |
| Hydrocephalus | - | - | 1.01  (0.25, 4.05) | 0.30  (0.02, 3.81) | 1.10  (0.02, 3.68) | 0.25  (0.63, 1.70) | 1.03  (0.63, 1.70) | 0.28  (0.03, 2.56) | - | - |
| Other disorders of the nervous system | 0.96  (0.79, 1.15) | 1.14  (0.85, 1.53) | 0.88  (0.73, 1.06) | 0.93  (0.67, 1.29) | 0.73  (0.68, 0.79) | 0.74  (0.58, 0.94) | 0.80  (0.69, 0.94) | 0.82  (0.61, 1.09) | 0.48  (0.33, 0.70) | 0.52  (0.33. 0.82) |
|  |  |  |  |  |  |  |  |  |  |  |
| IMMIGRANT MOTHER ONLY |  |  |  |  |  |  |  |  |  |  |
| **Any somatic condition** | 0.96  (0.94, 0.98) | 1.06  (1.02, 1.09) | 0.99  (0.89, 1.07) | 1.10  (1.00, 1.22) | 0.86  (0.83, 0.89) | 0.94  (0.90, 1.15) | 0.96  (0.88, 1.04) | 1.05  (0.96, 1.15) | 0.91  (0.85, 0.97) | 1.00  (0.93, 1.07) |
| **Infections** |  |  |  |  |  |  |  |  |  |  |
| Infections total | 1.03  (1.00, 1.06) | 1.14  (1.08, 1.20) | 0.99  (0.84, 1.17) | 1.14  (0.95, 1.36) | 0.85  (0.80, 0.90) | 0.96  (0.88, 1.05) | 1.11  (0.97, 1.28) | 1.24  (1.06, 1.43) | 1.05  (0.94, 1.17) | 1.18  (1.04, 1.33) |
| Intestinal infectious diseases | 0.90  (0.81, 1.02) | 0.95  (0.77, 1.17) | 0.47  (0.19, 1.12) | 0.49  (0.20, 1.21) | 0.87  (0.70, 1.09) | 0.88  (0.63, 1.22) | 0.80  (0.44, 1.44) | 0.81  (0.45, 1.52) | 1.14  (0.78, 1.65) | 1.16  (0.75, 1.79) |
| Tuberculosis | - | - |  |  | - | - |  |  |  |  |
| Other bacterial infections and sexually transmitted diseases | 1.16  (1.10, 1.23) | 1.30  (1.18, 1.42) | 0.91  (0.65, 1.28) | 1.08  (0.76, 1.55) | 0.99  (0.89, 1.10) | 1.18  (1.01, 1.37) | 1.27  (0.99. 1.63) | 1.45  (1.12, 1.88) | 1.11  (0.91 1.35) | 1.29  (1.03. 1.60) |
| Viral infections | 1.07  (1.00, 1.15) | 1.18  (1.04, 1.34) | 1.10  (0.78, 1.54) | 1.26  (0.87, 1.83) | 0.81  (0.70, 0.94) | 0.92  (0.75, 1.12) | 0.90  (0.63, 1.28) | 0.99  (0.69, 1.44) | 0.90  (0.69, 1.17) | 1.01  (0.75, 1.35) |
| Fungal and parasitic infections | 1.05  (0.97, 1.14) | 1.07  (0.92, 1.24) | 1.24  (0.86, 1.80) | 1.29  (0.86, 1.96) | 0.89  (0.76, 1.05) | 0.94  (0.74, 1.19) | 1.09  (0.75, 1.59) | 1.13  (0.75, 1.68) | 1.45  (1.13, 1.85) | 1.50  (1.12, 2.01) |
| Influenza and other acute lower respiratory tract infections | 0.97  (0.88, 1.08) | 1.10  (0.91, 1.32) | 0.69  (0.36, 1.32) | 0.79  (0.39, 1.57) | 0.78  (0.63, 0.97) | 0.84  (0.62, 1.15) | 0.94  (0.57, 1.56) | 1.03  (0.60, 1.76) | 1.06  (0.74, 1.51) | 1.17  (0.78, 1.76) |
| Infections of the skin and subcutaneous tissue | 0.99  (0.92,1.07) | 1.13  (0.99, 1.29) | 1.01  (0.69, 1.47) | 1.20  (0.80, 1.82) | 0.87  (0.75, 1.00) | 1.00  (0.81, 1.23) | 1.12  (0.81 1.56) | 1.28  (0.90, 1.81) | 1.08  (0.83, 1.38) | 1.23  (0.93, 1.64) |
| Infections of the musculoskeletal system and soft tissue | 0.80  (0.69, 0.93) | 0.86  (0.65, 1.13) | 1.13  (0.56, 2.26) | 1.19  (0.55, 2.59) | 0.61  (0.44, 0.84) | 0.62  (0.39, 0.97) | 0.87  (0.43, 1.74) | 0.89  (0.43, 1.88) | 0.73  (0.42, 1.29) | 1.75  (0.40, 1.43) |
| Urinary tract infections | 1.13  (1.04, 1.23) | 1.15  (0.98, 1.34) | 1.28  (0.82, 1.98) | 1.30  (0.81, 2.10) | 0.82  (0.68, 0.99) | 0.84  (0.65, 1.09) | 1.29  (0.86, 1.86) | 1.29  (0.86, 1.95) | 1.46  (1.11, 1.91) | 1.49  (1.09, 2.04) |
| Genital Infections | 0.97  (0.91, 1.05) | 1.16  (1.03, 1.32) | 1.29  (0.91, 1.83) | 1.65  (1.13, 2.41) | 0.92  (0.79, 1.04) | 1.11  (0.91, 1.35) | 0.93  (0.65, 1.32) | 1.10  (0.76, 1.60) | 1.07  (0.83, 1.37) | 1.30  (0.98, 1.72) |
| Infections of the CNS | 0.96  (0.67, 1.37) | 1.22  (0.64, 2.33) |  |  | 0.46  (0.17, 1.24) | 0.62  (0.18, 2.17) |  |  | - | - |
| **Non-infectious medical conditions** |  |  |  |  |  |  |  |  |  |  |
| Any non-infectious medical condition | 0.94  (0.92, 0.96) | 1.04  (1.00, 1.08) | 0.92  (0.83, 1.03) | 1.04  (0.93, 1.17) | 0.84  (0.81, 0.88) | 0.93  (0.88, 0.98) | 0.94  (0.85, 1.03) | 1.03  (0.93, 1.14) | 0.85  (0.79, 0.91) | 0.94  (0.86, 1.02) |
| Malignant neoplasms (outside CNS) | 1.05  (0.90, 1.23) | 1.13  (0.84, 1.52) | - | - | 0.78  (0.55, 1.22) | 0.87  (0.53, 1.43) | 0.51  (0.17, 1.60) | 0.56  (0.17, 1.79) | 0.57  (0.26, 1.28) | 0.63  (0.27, 1.49) |
| Benign neoplasms (outside CNS) | 1.01  (0.97, 1.05) | 1.01  (0.93, 1.08) | 0.97  (0.76, 1.23) | 0.97  (0.75, 1.25) | 0.83  (0.76, 0.90) | 0.84  (0.74, 0.95) | 1.05  (0.87, 1.28) | 1.06  (0.86, 1.30) | 0.83  (0.71, 0.98) | 0.84  (0.70, 1.00) |
| Blood diseases | 0.85  (0.76, 0.95) | 0.97  (0.80, 1.18) | 1.61  (1.05, 2.47) | 1.88  (1.15, 3.08) | 0.76  (0.61, 0.94) | 0.83  (0.61, 1.14) | 1.09  (0.68, 1.72) | 1.20  (0.73, 1.98) | 0.84  (0.60, 1.25) | 0.94  (0.61, 1.47) |
| Immune system disorders | 0.91  (0.66, 1.25) | 1.21  (0.69, 2.10) | - | - | - | - | - | - | - | - |
| Endocrine disorders | 0.97  (0.90, 1.04) | 1.14  (1.00, 1.29) | 1.25  (0.86, 1.83) | 1.54  (1.02, 2.32) | 0.92  (0.79, 1.06) | 1.07  (0.86, 1.32) | 1.46  (1.09, 1.96) | 1.68  (1.22, 2.31) | 0.93  (0.71, 1.23) | 1.09  (0.80, 1.49) |
| Malnutrition and problems with eating and feeding | 0.89  (0.79, 0.99) | 1.00  (0.82, 1.22) | 1.51  (1.01, 2.25) | 1.74  (1.08, 2.78) | 0.90  (0.74, 2.25) | 0.98  (0.73, 1.33) | 0.67  (0.37, 1.22) | 0.74  (0.40, 1.37) | 0.76  (0.50, 1.16) | 0.85  (0.53, 1.35) |
| Other nutritional deficiencies | 0.78  (0.64, 0.96) | 0.93  (0.66, 1.31) | 1.15  (0.57, 2.30) | 1.39  (0.61, 3.13) | 0.87  (0.63, 1.21) | 0.95  (0.57, 1.58) | 0.49  (0.16, 1.52) | 0.55  (1.17, 1.77) | 1.20  (0.69, 2.06) | 1.36  (0.71, 2.59) |
| Obesity and other hyperalimentation | 0.68  (0.62, 0.76) | 0.93  (0.78, 1.10) | 0.66  (0.36, 1.23) | 0.91  (0.48, 1.76) | 0.78  (0.56, 0.82) | 0.79  (0.60, 1.05) | 0.97  (0.64, 1.47) | 1.18  (0.76, 1.86) | 1.17  (0.88, 1.55) | 1.43  (1.02, 2.00) |
| Metabolic disorders | 0.84  (0.71, 0.99) | 1.01  (0.75, 1.35) | 0.77  (0.29, 2.04) | 0.97  (0.34, 2.78) | 0.60  (0.42, 0.88) | 0.74  (0.44, 1.22) | 1.14  (0.57, 2.28) | 1.36  (0.64, 2.86) | 0.40  (0.17, 0.96) | 0.48  (0.19, 1.23) |
| Visual impairment/blindness | 0.55  (0.31, 0.97) | 0.17  (0.03, 1.87) | - | - | 0.81  (0.34, 1.97) | 0.16  (0.02, 1.17) | - | - | - | - |
| Hearing impairment/deafness | 0.97  (0.87 1.09) | 1.18  (0.97, 1.44) | 1.00  (0.55, 1.80) | 1.29  (0.68, 2.46) | 0.73  (0.57, 0.93) | 0.91  (0.65, 1.26) | 0.57  (0.29, 1.15) | 0.69  (0.34, 1.42) | 1.01  (0.68, 1.50) | 1.25  (0.80, 1.94) |
| Diseases of the circulatory system outside of the CNS | 0.88  (0.82, 0.94) | 1.01  (0.90, 1.14) | 0.86  (0.60, 1.23) | 1.04  (0.70, 1.53) | 0.68  (0.59, 0.78) | 0.79  (0.65, 0.96) | 0.95  (0.70, 1.30) | 1.09  (0.79, 1.52) | 0.73  (0.56, 0.94) | 0.84  (0.63, 1.13) |
| Chronic lower respiratory disease (including asthma) | 0.89  (0.82, 0.97) | 1.04  (0.90, 1.20) | 1.07  (0.73, 1.57) | 1.34  (0.88, 2.05) | 0.68  (0.57, 0.81) | 0.83  (0.65, 1.05) | 1.27  (0.91, 1.72) | 1.50  (1.05, 2.15) | 1.05  (0.80, 1.38) | 1.26  (0.93, 1.72) |
| Diseases of the digestive system | 0.90  (0.86, 0.93) | 0.97  (0.90, 1.04) | 1.08  (0.89, 1.31) | 1.16  (0.94, 1.44) | 0.76  (0.70, 0.82) | 0.78  (0.69, 0.87) | 0.83  (0.69, 1.01) | 0.87  (0.71, 1.06) | 0.86  (0.75, 0.99) | 0.92  (0.80, 1.05) |
| Diseases of the skin and soft tissue | 0.99  (0.96, 1.02) | 1.05  (0.99, 1.11) | 1.05  (0.91, 1.22) | 1.14  (0.97, 1.35) | 0.96  (0.91, 1.02) | 1.03  (0.94, 1.12) | 0.97  (0.83, 1.12) | 1.03  (0.87, 1.20) | 0.91  (0.81, 1.03) | 0.97  (0.85, 1.11) |
| Diseases of the musculoskeletal system and connective tissue | 0.93  (0.90, 0.96) | 1.08  (1.02, 1.14) | 0.88  (0.74, 1.04) | 0.94  (0.88, 1.26) | 0.75  (0.71, 0.80) | 0.86  (0.78, 0.94) | 0.93  (0.81, 1.08) | 1.06  (0.91, 1.24) | 0.86  (0.77, 0.96) | 0.99  (0.87, 1.12) |
| Urinary tract diseases | 0.91  (0.85, 0.98) | 1.06  (0.92, 1.21) | 1.13  (0.78, 1.63) | 1.32  (0.88, 1.98) | 0.69  (0.58, 0.81) | 0.75  (0.60, 0.94) | 1.05  (0.75, 1.47) | 1.16  (0.81, 1.67) | 0.84  (0.64, 1.12) | 0.94  (0.69 1.29) |
| Genital diseases and disorders of breast | 0.97  (0.94, 1.00) | 1.08  (0.95, 1.24) | 1.03  (0.87, 1.22) | 0.99  (0.86, 1.14) | 0.86  (0.81, 0.92) | 0.80  (0.72, 0.89) | 1.04  (0.90, 1.20) | 0.79  (0.69, 0.90) | 0.92  (0.82, 1.03) | 1.10  (0.94, 1.30nn) |
| **Non-infectious neurological conditions** |  |  |  |  |  |  |  |  |  |  |
| Neurological conditions total | 0.92  (0.88, 0.96) | 1.06  (0.98, 1.14) | 0.69  (0.53, 0.90) | 0.81  (0.62, 1.08) | 0.79  (0.73, 0.90) | 0.88  (0.77, 0.99) | 0.80  (0.64, 1.00) | 0.89  (0.71, 1.12) | 1.02  (0.88, 1.18) | 1.15  (0.98, 1.35) |
| Sleeping disorders | 0.96  (0.88, 1.04) | 1.11  (0.96, 1.28) | 0.61  (0.34, 1.07) | 0.71  (0.39, 1.28) | 1.05  (0.91, 1.22) | 1.14  (0.91, 1.43) | 1.01  (0.68, 1.48) | 1.11  (0.74, 1.67) | 1.23  (0.95, 1.60) | 1.37  (1.02, 1.85) |
| Neoplasms of the CNS (malignant and benign) | 1.43  (1.06, 1.93) | 1.48  (0.85, 2.59) | - | - | 1.10  (0.57, 2.11) | 1.16  (0.46, 2.91) | - | - | 2.24  (0.93, 5.40) | 2.36  (0.82, 6.77) |
| Cerebrovascular diseases | 1.02  (0.79, 1.33) | 1.26  (0.79, 2.00) | - | - | 0.67  (0.36, 1.25) | 0.84  (0.47, 1.93) | - | - | 0.99  (0.37, 2.63) | 1.23  (0.41, 3.68) |
| Epilepsy | 1.06  (0.91, 1.23) | 1.25  (0.95, 1.65) | 0.56  (0.17, 1.75) | 0.70  (0.21, 2.27) | 0.65  (0.45, 0.94) | 0.76  (0.46, 1.24) | 1.52  (0.82, 2.82) | 1.76  (0.90, 3.45) | 1.71  (1.10, 2.65) | 2.01  (1.19, 3.39) |
| Headache conditions (including migraine) | 0.86  (0.80, 0.91) | 1.00  (0.89, 1.12) | 0.76  (0.54, 1.07) | 0.90  (0.62, 1.32) | 0.65  (0.57, 0.74) | 0.73  (0.60, 0.88) | 0.57  (0.40, 0.83) | 0.64  (0.44, 0.95) | 0.91  (0.73, 1.14) | 1.03  (0.80, 1.33) |
| Cerebral palsy | 1.28  (0.84, 1.96) | 2.43  (1.25, 4.70) | - | - | 1.47  (0.66,3.28) | 1.14  (0.78, 11.05) | - | - | 1.75  (0.44, 7.03) | 3.86  (0.82, 18.17) |
| Hydrocephalus | 1.32  (0.85, 2.07) | 1.04  (0.40, 2.67) | - | - | 0.74  (0.24, 2.31) | 0.71  (0.23, 2.22) | - | - | - | - |
| Other disorders of the nervous system | 0.98  (0.91, 1.04) | 1.18  (1.05, 1.33) | 0.71  (0.46, 1.10) | 0.90  (0.57, 1.44) | 0.72  (0.63, 0.83) | 0.86  (0.70, 1.06) | 0.78  (0.54, 1.12) | 0.93  (0.63, 1.35) | 0.96  (0.75, 1.23) | 1.16  (0.88, 1.52) |
|  |  |  |  |  |  |  |  |  |  |  |
| IMMIGRANT FATHER ONLY |  |  |  |  |  |  |  |  |  |  |
| **Any somatic condition** | 1.01  (0.99, 1.03) | 1.03  (1.00, 1.06) | 1.15  (1.06, 1.24) | 1.14  (1.04, 1.24) | 1.05  (1.01, 1.09) | 1.05  (1.00, 1.09) | 1.04  (1.00, 1.08) | 1.05  (1.00, 1.10) | 1.13  (1.08, 1.21) | 1.15  (1.08, 1.23) |
| **Infections** |  |  |  |  |  |  |  |  |  |  |
| Infections total | 1.08  (1.05, 1.11) | 1.03  (0.98, 1.09) | 1.31  (1.15, 1.49) | 1.22  (1.06, 1.40) | 1.23  (1.16, 1.30) | 1.16  (1.07, 1.25) | 1.31  (1.23, 1.40) | 1.24  (1.14, 1.35) | 1.39  (1.27, 1.52) | 1.31  (1.19, 1.45) |
| Intestinal infectious diseases | 0.99  (0.89, 1.11) | 1.08  (0.90, 1.29) | 1.16  (0.72, 1.86) | 1.22  (0.73, 2.05) | 1.10  (0.90, 1.35) | 1.17  (0.62, 1.10) | 0.83  (0.62, 1.10) | 0.90  (0.64, 1.27) | 1.16  (0.83, 1.62) | 1.25  (0.88, 1.82) |
| Tuberculosis | - | - |  |  | - | - | - | - |  |  |
| Other bacterial infections and sexually transmitted diseases | 1.15  (1.09, 1.21) | 1.08  (0.99, 1.19) | 1.12  (0.87, 1.45) | 1.07  (0.81, 1.40) | 1.22  (1.10, 1.36) | 1.16  (1.01, 1.33) | 1.49  (1.33, 1.67) | 1.40  (1.20, 1.62) | 1.79  (1.56, 2.02) | 1.69  (1.43, 2.00) |
| Viral infections | 1.04  (0.97, 1.11) | 1.05  (0.94, 1.19) | 1.26  (0.94, 1.69) | 1.28  (0.93, 1.76) | 1.11  (0.96, 1.26) | 1.10  (0.94, 1.33) | 1.21  (1.03, 1.41) | 1.22  (1.00, 1.49) | 1.18  (0.95, 1.46) | 1.19  (0.94, 1.52) |
| Fungal and parasitic infections | 1.10  (1.02, 1.20) | 0.97  (0.84, 1.11) | 1.58  (1.16, 2.15) | 1.37  (0.97, 1.93) | 1.45  (1.26, 1.66) | 1.27  (0.95 1.49) | 1.38  (1.16, 1.64) | 1.18  (0.95, 1.48) | 1.51  (1.20, 1.88) | 1.31  (1.01, 1.70) |
| Influenza and other acute lower respiratory tract infections | 0.99  (0.89, 1.10) | 0.84  (0.70, 1.02) | 1.50  (1.01, 2.22) | 1.13  (0.73, 1.76) | 1.07  (0.88, 1.31) | 0.84  (0.64, 1.09) | 1.11  (0.87, 1.40) | 0.88  (0.64, 1.19) | 1.12  (0.81, 1.54) | 0.89  (0.62, 1.29) |
| Infections of the skin and subcutaneous tissue | 1.08  (1.00, 1.15) | 1.02  (0.90, 1.14) | 1.55  (1.18, 2.04) | 1.39  (1.03, 1.88) | 1.50  (1.33, 1.69) | 1.37  (1.16, 1.61) | 1.60  (1.39, 1.84) | 1.46  (1.22, 1.59) | 1.37  (1.12, 1.68) | 1.26  (1.00, 1.59) |
| Infections of the musculoskeletal system and soft tissue | 0.91  (0.79, 1.05) | 0.86  (0.67, 1.10) | 0.71  (0.34, 1.50) | 0.61  (0.28, 1.35) | 0.99  (0.76, 1.30) | 0.87  (0.61, 1.25) | 0.64  (0.43, 0.96) | 0.58  (0.36, 0.93) | 1.20  (0.80, 1.79) | 1.08  (0.67, 1.73) |
| Urinary tract infections | 1.27  (1.07, 1.26) | 1.07  (0.93, 1.24) | 1.28  (0.88, 1.86) | 1.16  (0.77, 1.75) | 1.41  (1.21, 1.64) | 1.29  (1.05, 1.58) | 1.78  (1.51, 2.09) | 1.61  (1.29, 2.01) | 1.80  (1.44, 2.25) | 1.64  (1.26, 2.13) |
| Genital Infections | 1.12  (1.05, 1.19) | 1.09  (0.97, 1.22) | 1.41  (1.06, 1.87) | 1.31  (0.96, 1.79) | 1.25  (1.11, 1.42) | 1.18  (1.00, 1.40) | 1.28  (1.10, 1.48) | 1.22  (1.01, 1.48) | 1.39  (1.13, 1.68) | 1.31  (1.05, 1.65) |
| Infections of the CNS | 1.02  (0.76, 1.49) | 1.10  (0.62, 1.98) | - | - | 0.76  (0.34, 1.71) | 0.78  (0.29, 2.10) | 1.12  (0.50, 2.49) | 1.15  (0.42, 3.18) | - | - |
| **Non-infectious medical conditions** |  |  |  |  |  |  |  |  |  |  |
| Any non-infectious medical condition | 0.98  (0.97, 1.00) | 1.02  (0.99, 1.05) | 1.14  (1.04, 1.25) | 1.15  (1.05, 1.27) | 1.03  (0.99, 1.07) | 1.04  (0.99, 1.10) | 0.98  (0.94, 1.03) | 1.01  (0.95, 1.07) | 1.07  (1.00, 1.13) | 1.09  (1.02, 1.17) |
| Malignant neoplasms (outside CNS) | 0.91  (0.77, 1.08) | 0.87  (0.64, 1.17) | - | - | 0.69  (0.47, 1.03) | 0.65  (0.40, 1.07) | 1.07  (0.73, 1.56) | 1.00  (0.61, 1.65) | 0.53  (0.25, 1.10) | 0.49  (0.22, 1.10) |
| Benign neoplasms (outside CNS) | 1.03  (0.99, 1.07) | 1.06  (0.99, 1.14) | 1.03  (0.85, 1.24) | 1.09  (0.88, 1.33) | 1.01  (0.93, 1.09) | 1.06  (0.95, 1.17) | 0.82  (0.74, 0.91) | 0.86  (0.75, 0.98) | 0.92  (0.80, 1.06) | 0.96  (0.83, 1.12) |
| Blood diseases | 0.84  (0.75, 0.93) | 0.90  (0.75, 1.08) | 1.13  (0.73, 1.75) | 1.15  (0.71, 1.86) | 1.20  (1.00, 1.44) | 1.23  (0.96, 1.59) | 0.96  (0.75, 1.22) | 1.02  (0.75, 1.39) | 0.77  (0.53, 1.12) | 0.82  (0.54, 1.23) |
| Immune system disorders | 1.21  (0.12, 1.59) | 1.20  (0.74, 1.95) | - | - | 0.76  (0.38, 1.53) | 0.74  (0.32, 1.72) | 0.96  (0.46, 2.02) | 0.94  (0.38, 2.33) | 1.30  (0.54, 3.12) | 1.26  (0.46, 3.45) |
| Endocrine disorders | 1.05  (0.98, 1.12) | 1.10  (0.98, 1.25) | 1.46  (1.09, 1.95) | 1.47  (1.07, 2.03) | 1.32  (1.17, 1.50) | 1.35  (1.13, 1.61) | 0.95  (0.80, 1.14) | 1.00  (0.80 1.24) | 1.27  (1.02, 1.57) | 1.31  (1.03, 1.68) |
| Malnutrition and problems with eating and feeding | 0.98  (0.88, 1.09) | 1.07  (0.90, 1.28) | 1.22  (0.80, 1.87) | 1.28  (0.80, 2.04) | 0.93  (0.75, 1.15) | 0.97  (0.74, 1.28) | 0.99  (0.77, 1.28) | 1.07  (0.79, 1.47) | 1.17  (0.86, 1.61) | 1.26  (0.88, 1.80) |
| Other nutritional deficiencies | 0.89  (0.74, 1.08) | 0.76  (0.55, 1.05) | 0.80  (0.33, 1.91) | 0.56  (0.22, 1.44) | 1.76  (1.36, 2.29) | 1.30  (0.87, 1.94) | 1.52  (1.07, 2.15) | 1.17  (0.73, 1.88) | 1.26  (0.76, 2.10) | 0.98  (0.55, 1.77) |
| Obesity and other hyperalimentation | 0.71  (0.64, 0.78) | 0.72  (0.60, 0.85) | 1.47  (1.06, 2.04) | 1.16  (0.80, 1.70) | 1.02  (0.81, 1.20) | 0.84  (0.64, 1.11) | 0.92  (0.75, 1.13) | 0.84  (0.64, 1.11) | 1.40  (1.11, 1.77) | 1.26  (0.95, 1.67) |
| Metabolic disorders | 0.91  (0.77, 1.06) | 0.97  (0.74, 1.27) | 0.94  (0.45, 1.96) | 0.97  (0.43, 2.15) | 0.81  (0.58, 1.14) | 0.84  (0.55, 1.30) | 0.66  (0.42, 1.03) | 0.70  (0.41, 1.20) | 0.91  (0.54, 1.54) | 0.96  (0.53, 1.73) |
| Visual impairment/blindness | 0.77  (0.47 1.24) | 0.68  (0.29, 1.62) | - | - | 0.55  (0.18, 1.71) | 0.43  (0.10, 1.75) | 1.63  (0.73, 3.63) | 1.32  (0.40, 4.40) | - | - |
| Hearing impairment/deafness | 0.97  (0.87, 1.08) | 0.92  (0.76, 1.22) | 0.42  (0.27, 1.09) | 0.48  (0.23, 1.00) | 0.96  (0.77, 1.19) | 0.86  (0.64, 1.16) | 0.77  (0.57, 1.04) | 0.71  (0.49, 1.02) | 0.87  (0.59, 1.27) | 0.80  (0.52. 1.23) |
| Diseases of the circulatory system outside of the CNS | 0.87  (0.82, 0.93) | 0.88  (0.79, 0.99) | 1.04  (0.79, 1.39) | 1.02  (0.75, 1.39) | 0.91  (0.80, 1.03) | 0.89  (0.75, 1.06) | 0.92  (0.79, 1.08) | 0.92  (0.76, 1.12) | 0.75  (0.59, 0.95) | 0.75  (0.58, 0.97) |
| Chronic lower respiratory disease (including asthma) | 1.00  (0.92, 1.08) | 1.08  (0.95, 1.23) | 0.94  (0.64, 1.37) | 1.03  (0.69, 1.55) | 1.04  (0.89, 1.21) | 1.14  (0.93, 1.39) | 1.19  (0.97, 1.39) | 1.28  (1.03, 1.60) | 1.21  (0.96, 1.53) | 1.33  (1.02, 1.74) |
| Diseases of the digestive system | 0.98  (0.95, 1.02) | 1.03  (0.96, 1.09) | 1.24  (1.06, 1.44) | 1.22  (1.03, 1.44) | 1.13  (1.06, 1.21) | 1.13  (1.03, 1.24) | 0.93  (0.85, 1.01) | 0.95  (0.85, 1.06) | 1.15  (1.03, 1.28) | 1.17  (1.03, 1.33) |
| Diseases of the skin and soft tissue | 0.98  (0.95, 1.01) | 0.97  (0.92, 1.03) | 1.14  (1.00, 1.31) | 1.11  (0.98, 1.29) | 1.13  (1.07, 1.20) | 1.10  (1.02, 1.20) | 1.03  (0.96, 1.11) | 1.02  (0.92, 1.12) | 1.06  (0.96, 1.18) | 1.05  (0.93, 1.17) |
| Diseases of the musculoskeletal system and connective tissue | 0.99  (0.96, 1.02) | 1.03  (0.98, 1.08) | 1.17  (1.03, 1.33) | 1.14  (0.99, 1.31) | 1.06  (1.00, 1.12) | 1.05  (0.97, 1.13) | 1.05  (0.98, 1.12) | 1.06  (0.97, 1.16) | 1.05  (0.96, 1.16) | 1.06  (0.95, 1.18) |
| Urinary tract diseases | 0.95  (0.88, 1.02) | 1.00  (0.89, 1.13) | 0.93  (0.66, 1.31) | 0.91  (0.62, 1.32) | 1.11  (0.97, 1.27) | 1.10  (0.92, 1.32) | 0.99  (0.83, 1.17) | 1.02  (0.82, 1.27) | 1.20  (0.97, 1.49) | 1.22  (0.96, 1.56) |
| Genital diseases and disorders of breast | 1.01  (0.98, 1.04) | 1.06  (1.00, 1.11) | 1.28  (1.12, 1.45) | 1.30  (0.96, 1.13) | 1.01  (0.96, 1.08) | 1.04  (0.96, 1.13) | 1.00  (0.93, 1.07) | 1.04  (0.95 1.14) | 1.12  (1.02, 1.23) | 1.16  (1.04, 1.29) |
| **Non-infectious neurological conditions** |  |  |  |  |  |  |  |  |  |  |
| Neurological conditions total | 1.00 (0.96, 1.04) | 1.06  (0.99, 1.14) | 1.18  (0.99, 1.41) | 1.18  (0.97, 1.42) | 1.08  (1.00, 1.17) | 1.08  (0.98, 1.20) | 1.02  (0.93, 1.13) | 1.07  (0.95, 1.20) | 1.13  (1.00, 1.29) | 1.17  (1.02, 1.35) |
| Sleeping disorders | 1.02  (0.96, 1.11) | 1.04  (0.91, 1.19) | 1.55  (1.16, 2.09) | 1.43  (1.03, 1.99) | 1.25  (1.09, 1.44) | 1.18  (0.97, 1.43) | 1.42  (1.21, 1.66) | 1.38  (1.11, 1.71) | 1.29  (1.03, 1.63) | 1.25  (0.96, 1.63) |
| Neoplasms of the CNS (malignant and benign) | 1.05  (0.74, 1.49) | 0.93  (0.49, 1.75) | - | - | 1.07  (0.53, 2.14) | 0.92  (0.36, 2.37) | 0.78  (0.29, 2.07) | 0.66  (0.20, 2.46) | - | - |
| Cerebrovascular diseases | 0.90  (0.68, 1.18) | 0.92  (0.70, 1.22) | - | - | 1.24  (0.77, 1.99) | 1.23  (0.78, 1.98) | 0.63  (0.28, 1.40) | 0.70  (0.33, 1.46) | 1.38  (0.66, 2.90) | 1.52  (0.76, 3.04) |
| Epilepsy | 1.11  (0.96, 1.29) | 0.92  (0.70, 1.22) | 2.10  (1.24, 3.54) | 1.54  (0.84, 2.80) | 1.23  (0.92, 1.64) | 0.93  (0.63, 1.38) | 1.24  (0.88, 1.76) | 0.96  (0.61, 1.50) | 1.58  (1.04, 2.40) | 1.23  (0.75, 2.02) |
| Headache conditions (including migraine) | 0.99  (0.93, 1.05) | 1.11  (1.00, 1.22) | 1.08  (0.83, 1.41) | 1.15  (0.86, 1.53) | 0.98  (0.87, 1.10) | 1.04  (0.89, 1.21) | 0.73  (0.62, 0.86) | 0.81  (0.66, 0.98) | 1.07  (0.88, 1.29) | 1.16  (0.94, 1.44) |
| Cerebral palsy | 0.97  (0.60, 1.57) | 0.51  (0.18, 1.49) | - | - | 0.78  (0.25, 2.43) | 0.36  (0.08, 1.71) | 1.90 (0.79, 4.59) | 0.86  (0.21, 3.54) | 1.43  (0.36, 5.72) | 0.68  (0.12, 3.85) |
| Hydrocephalus | 0.59  (0.30, 1.13) | 0.48  (0.16, 1.50) | - | - | - | - | 2.44  (1.09, 5.45) | 1.88  (0.97, 7.57) | 3.02  (1.13, 8.08) | 2.37  (0.56, 10.06) |
| Other disorders of the nervous system | 0.99  (0.93, 1.06) | 1.00  (0.90, 1.13) | 1.10  (0.81, 1.48) | 1.03  (0.74, 1.42) | 1.05  (0.93, 1.20) | 1.00  (0.85 1.19) | 1.05  (0.90, 1.22) | 1.03  (0.84, 1.25) | 0.98  (0.79, 1.23) | 0.96  (0.75, 1.23) |
| HR not calculated if N≤5 (for cerebral palsy among those with an immigrant mother only, HR could not be calculated for two regions of origin although N >5 & <10). | | | | | | | | | | |
